# Mitigation of Coral Reef Warming Across the Central Pacific by the Equatorial Undercurrent: A Past and Future Divide

**DOI:** 10.1038/srep21213

**Published:** 2016-02-16

**Authors:** Kristopher B. Karnauskas, Anne L. Cohen, Jamison M. Gove

**Affiliations:** 1 Cooperative Institute for Research in Environmental Sciences, and Department of Atmospheric and Oceanic Sciences, University of Colorado, Boulder, Colorado, USA; 2 Department of Geology and Geophysics, Woods Hole Oceanographic Institution, Woods Hole, Massachusetts, USA; 3 Ecosystems and Oceanography Program, NOAA Pacific Islands Fisheries Science Center, Honolulu, Hawaii, USA

## Abstract

Global climate models (GCMs) predict enhanced warming and nutrient decline across the central tropical Pacific as trade winds weaken with global warming. Concurrent changes in circulation, however, have potential to mitigate these effects for equatorial islands. The implications for densely populated island nations, whose livelihoods depend on ecosystem services, are significant. A unique suite of *in situ* measurements coupled with state-of-the-art GCM simulations enables us to quantify the mitigation potential of the projected circulation change for three coral reef ecosystems under two future scenarios. Estimated historical trends indicate that over 100% of the large-scale warming to date has been offset locally by changes in circulation, while future simulations predict a warming mitigation effect of only 5–10% depending on the island. The pace and extent to which GCM projections overwhelm historical trends will play a key role in defining the fate of marine ecosystems and island communities across the tropical Pacific.

The central tropical Pacific Ocean hosts some of the world’s most productive coral reef ecosystems and yields essential ecosystem services to millions of people across dozens of nations through coastal protection, fisheries, and tourism. However, this region is also among the most vulnerable to the effects of global warming. Here, global climate models (GCMs) project enhanced warming of the sea surface by ~3 °C by the end of this century, driven by a weakening of the easterly trade winds and equatorial upwelling^1^,^2^. This rate of warming, and the concomitant depletion of surface nutrients, could have potentially devastating effects on the region’s marine ecosystems and the human communities that depend on them^3^.

GCMs simulate the large-scale state of the ocean and climate system but are unaware of small coral reef islands and atolls (islands hereafter). Even state-of-the-art GCMs do not resolve islands much smaller than 100 km across, since the horizontal resolution of the ocean components of CMIP5 models typically range from 0.2–2°^4^. Many of the central Pacific islands, several with substantial human communities (*e.g.*, Tarawa, Republic of Kiribati), are an order of magnitude smaller than this. While this “blindness” is of little consequence in the open ocean or near islands situated within a relatively stagnant ocean environment, it has critical implications for the equatorial Pacific where islands interact with swift zonal currents, transforming the local oceanography in ways that cannot be captured by GCMs^5^,^6^.

The Pacific Equatorial Undercurrent (EUC) travels eastward along the equator at 50–200 m depth^7^. When the EUC encounters equatorial islands, topographic upwelling and mixing delivers cool, nutrient rich water to their western sides, creating a cross-island temperature gradient (cooler on the west, warmer on the east) modulated by EUC strength^5^,^8^. The same GCMs that predict enhanced warming in the tropical Pacific also project strengthening of the EUC^5^,^9^,^10^. Because EUC velocity is linked to island SST through topographic upwelling, a future strengthening of the EUC could mitigate surface warming and associated ecosystem impacts for equatorial reefs.

Previously, we used high-resolution satellite data to estimate the mean cross-island temperature gradient at the Gilbert Islands (Republic of Kiribati, 174°E, 0°N; order 10 km), and a high-resolution numerical ocean model to estimate its sensitivity to EUC strength^5^. Combining these estimates with future EUC changes projected by Coupled Model Intercomparison Project 3 (CMIP3) GCMs yielded in an estimated warming mitigation at the Gilberts of 25 ± 9% per century^5^. Here we exploit a new and unique suite of *in situ* measurements to constrain the actual cross-island temperature gradient at three important central Pacific sites, each smaller than the usable resolution of infrared or microwave satellite measurements and beyond the reasonable capability of most ocean and climate models to resolve. Further, the latest generation of GCM simulations (CMIP5) and analysis of long-term estimated historical trends in EUC strength^11^ significantly improves our ability to quantify the EUC’s mitigation potential for all four sites (Gilberts, Baker, Howland, and Jarvis) under a range of future scenarios describing temperature and circulation change.

## Results

### Observations and empirical model development

The U.S. Pacific Remote Islands Marine National Monument (PRIMNM), which includes Howland, Baker and Jarvis, was established in 2009 by Presidential Proclamation and accounts for the broadest collection of marine protected areas under U.S. jurisdiction. Loggers deployed by NOAA’s Coral Reef Ecosystem Division (CRED) on the west and east sides of the islands recorded reef-level temperatures continuously over the past 10 years (Supplementary Data Table S1). These data allow us to compute the cross-island temperature gradient (δT) directly from observations, analyze its co-variability with the observed EUC, develop well-constrained empirical models, and apply them to the latest generation of GCM projections^4^ as well as observed trends.

**Figure 1:**
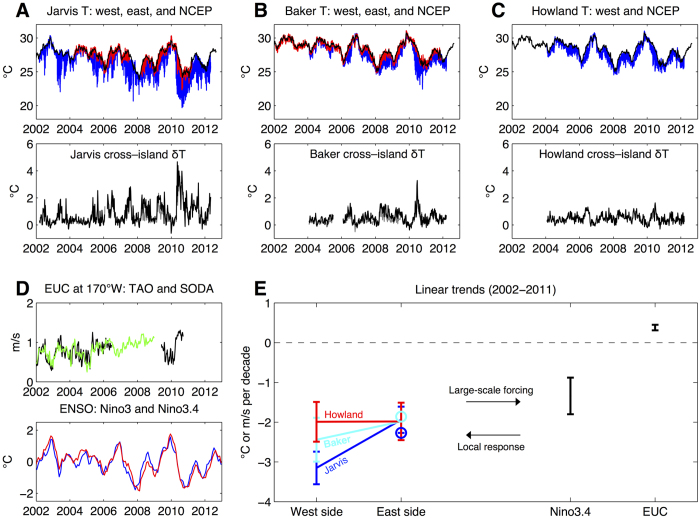
Summary of observations. (**A**) Top: logger measured temperature on the west (blue) and east (red) sides of Jarvis Island (160.02°W, 0.38°S), native sampling resolution, and NCEP SST^21^ from the nearest grid cell (black), weekly averaged (°C). Bottom: cross-island temperature gradient (δT) at Jarvis based on the west logger contrasted with the east logger (gray) and NCEP SST (black), weekly averaged (°C). (**B**,**C**) As in (**A**) but for Baker (176.49°W, 0.19°N) and Howland (176.62°W, 0.81°N) Islands. (**D**) Top: EUC velocity at 170°W measured by TAO (black) and estimated by SODA (green), weekly averaged (m/s). Bottom row: Commonly used ENSO indices Nino3 (blue) and Nino3.4 (red), monthly averaged (°C). (**E**) Linear trends in temperature measured on the west and east sides of each island and in the Nino3.4 region (°C per decade) over the period 2002–2011*. NCEP SST is used for the east side; circles indicate equivalent trends based on east loggers at Jarvis and Baker. Also indicated is the trend in the merged 170°W EUC record over the same period*. Error bars indicate 95% confidence intervals of the trend. *Trends at Baker and Howland are computed beginning with 2004, prior to which the west loggers were not deployed at those islands. The EUC trend is computed over the period ending 2010, after which neither SODA nor 170°W TAO zonal velocity data are available.

The decreasing mean and standard deviation of δT from Jarvis (0.83 ± 0.80 °C) to Baker (0.54 ± 0.45 °C) to Howland (0.48 ± 0.32 °C) is consistent with the geographic context of each island relative to the mean structure of the EUC and thermocline (Fig. 1A–C). Jarvis is situated at 160°W where, on average, the EUC is ~0.2 m/s faster and the thermocline ~20 m shallower than at Baker and Howland (176°W)^7^. Moreover, Howland is 69 km further north of the equator than Baker, thus further from the core of the EUC (average difference ~0.1 m/s). Despite differences in mean δT and its amplitude of variability at each island, δT is similarly and significantly correlated with the strength of the EUC (Fig. 1D; correlations of 0.59, 0.60, and 0.50 at Jarvis, Baker, and Howland, respectively).

Linear trends over the period of measurement reflect the strong control of the EUC on δT. While the broader central equatorial Pacific (Nino3.4) experienced a cooling trend of −1.34 ± 0.46 °C per decade, which is not significantly different from trends on the east side of each island (~2 °C per decade), the west-side cooling trends were stronger, especially at Baker and Jarvis (Fig. 1E). The cooling trend on the west side of Jarvis was, in fact, significantly stronger than that on its east side (−3.15 ± 0.41 °C per decade compared to −1.94 ± 0.33 °C per decade). A simple explanation for these cross-island trends and their inter-island variability is that the EUC was also strengthening over this period by 0.38 ± 0.07 m/s per decade (Fig. 1D), leading to stronger topographic upwelling and enhanced cooling on the west sides of the islands that stand directly in its path.

**Figure 2:**
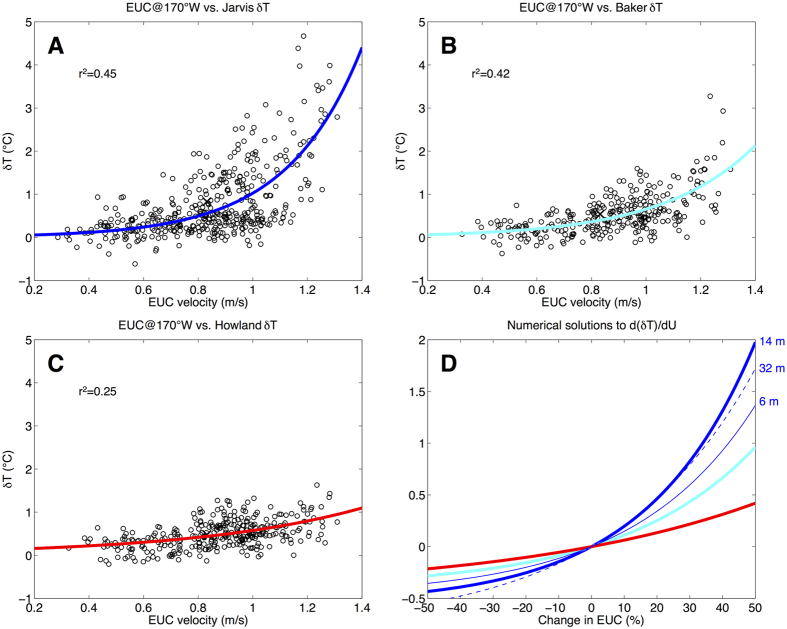
Empirical model development. (**A**) Scatter plot of EUC velocity at 170°W (m/s) and (δT) at Jarvis (°C) with an exponential model estimated by nonlinear least squares regression. (**B**,**C**) As in (**A**) but for Baker and Howland Islands. Adjusted r-squared values and mean values (“x” marks) are provided in each panel. (**D**) Solutions to the nonlinear regression models shown in (**A**–**C**) expressed as change in δT (°C) as a function of change in EUC relative to the time mean EUC (%). Solutions to models estimated using δT based on logger temperatures from alternative depths at west Jarvis (thin lines) indicate sensitivity to depth (note, however, that only 4–5 years of data are available from the alternative west Jarvis loggers).

Records of δT are paired with a merged record of EUC velocity^12^,^13^ (see Methods and Supplementary Data Table S1) to develop empirical models by nonlinear regression (Fig. 2A–C, S4). Solutions to the models predict the change in δT for a given percent change in EUC velocity (Fig. 2D). For example, a 20% strengthening of the EUC would result in a 0.5 °C increase in δT at Jarvis (*i.e.*, from 0.8 °C to 1.3 °C). The model for Baker would yield roughly half the increase in δT for the same increase in EUC velocity, and half again for Howland. An EUC-related change in δT would be manifest as a change in temperature on the west side of the island. The empirical models describing the dependence of δT on EUC velocity can therefore be applied to GCM future projections of EUC strength and weighed against the projected changes in sea surface temperature (SST) for the region (*i.e.*, oblivious to island influences) in the same GCM simulations. The result can effectively be interpreted as a “mitigation effect” on the west sides of the islands.

### Application to GCM projections

**Figure 3:**
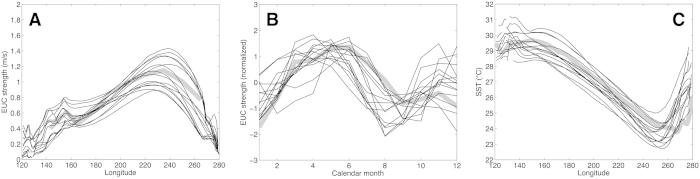
Climate model fidelity in the equatorial Pacific. (**A**) Profiles along the equator of the maximum EUC velocity (m/s), (**B**) mean seasonal cycles of maximum EUC velocity at 170°W (normalized), and (**C**) as in (**A**) but for equatorial SST (°C). The 14 models shown (thin black lines) are those that passed the screening process for reasonable simulation of the EUC magnitude, zonal structure, and seasonality (see Methods and Fig. S5). Provided in A–B are observational estimates of the mean EUC and its seasonal cycle^7^ and equatorial SST^21^.

The SST and zonal velocity output fields of 35 GCMs from the CMIP5 archive (see Supplementary Data Table S2) were analyzed following a screening process for reasonable simulation of the magnitude, zonal structure, and seasonality of the EUC (see Methods and Fig. S5). Although the analogy is not perfect, both the seasonal cycle and long-term climate change are essentially a response to changes in radiative forcing, so simulating a realistic seasonal cycle is a minimum core requirement for placing confidence in climate change projections. It should also be noted that a realistic simulation of the EUC is a particularly rigorous target because its mean properties are determined by integrating several coupled climate processes including the trade winds, the large-scale thermocline structure, and vertical mixing of momentum; many CMIP3 models had difficulty capturing the magnitude and zonal structure^14^. The remaining 14 models show a very reasonable spread about the observed EUC magnitude, zonal structure and seasonality (Fig. 3A,B); those models capture well the zonal structure of the equatorial SST field as well (Fig. 3C).

**Figure 4:**
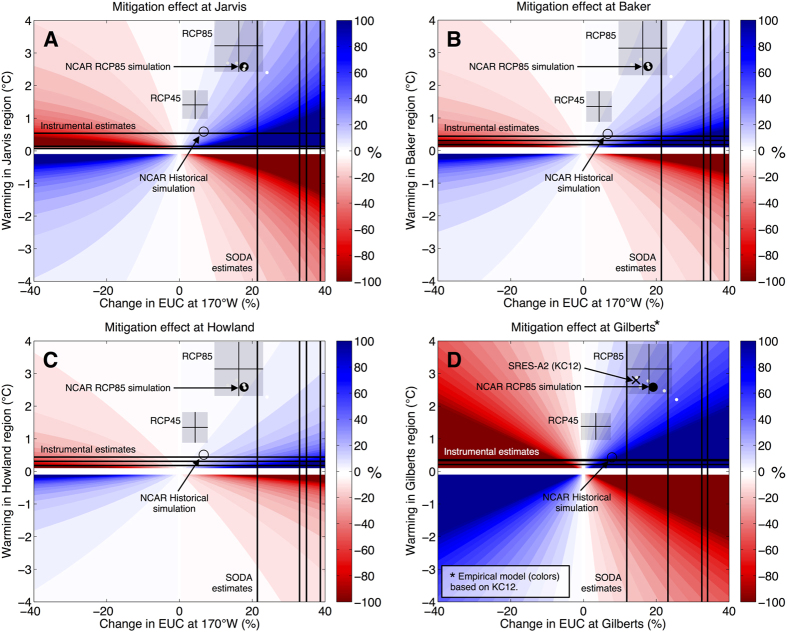
Historical and future mitigation effect. (**A**) Colors: Mitigation effect on the west side of Jarvis Island (%) as a function of the predicted changes in EUC at 170°W (%) and SST for the region surrounding Jarvis Island (°C). (“Region” is defined as the scale of a grid cell of a global climate model that does not include such small islands.) Vertical lines: Four estimates of the historical trends in EUC at 170°W based on the SODA ocean reanalysis: v.2.2.4/1871-2008, v.2.2.4/1910-2008, v.2.2.6/1871-2008, and v.2.2.6/1910-2008 (%/century). Horizontal lines: Historical trends in regional SST over the period 1870–2012 (°C/century) based on three widely use instrumental data sets. Open circle: Simulated trend (per century) in EUC and SST based on the Historical experiments by the NCAR global climate model spanning 1870–2004. Closed black circle: As in open circle but for projections based on RCP8.5 experiments spanning 2006–2100. Small white dots indicate the spread of individual ensemble runs of the NCAR model. Transparent boxes indicate the CMIP5 multi-model mean projections for RCP4.5 and RCP8.5 as labeled, including +/− 2 standard errors inter-model spread about the multi-model mean trends. The multi-model mean is comprised of the 14 models passing the screening for realistic EUC strength, zonal structure, and seasonality (see Materials and Methods). (**B**,**C**) As in (**A**) but for Baker and Howland Islands. (**D**) As in (**A**–**C**) but for the Gilbert Islands and based on the model of KC12 rather than *in situ* data. The “x” symbol marks the Gilberts projection based on CMIP3 models^5^.

The empirical models as expressed in Fig. 2D (or as ordinary differential equations in Fig. S4) are solved for a range of changes in EUC velocity (ΔU) and compared to a range of large-scale changes in SST (ΔT) from historical estimates and CMIP5 model projections (Fig. 4). For example, under the RCP8.5 scenario as prescribed by IPCC AR5, the NCAR CCSM4 climate model^15^ predicts a warming at Jarvis of 2.6 °C per century (see Methods). The same experiment also predicts a 13–24% (17.7% ensemble mean) per century strengthening of the EUC, which increases δT (cools west-side SST) by an amount equivalent to 10–25% (15% ensemble mean) of the 2.6 °C warming. The complete ensemble mean projection based on all 14 screened CMIP5 models places the mitigation effect for Jarvis at 11% (3.2 °C warming and 16.3% increase in EUC strength). Note that the projected EUC trend grows as the screening criteria become more stringent (Table 1). Results for Baker and Howland are qualitatively similar but yield a smaller mitigation effect due to the weaker sensitivity of their δT to EUC velocity than at Jarvis given the physical mechanisms previously discussed. Note that an additional robust aspect of the projected response of the equatorial Pacific to anthropogenic forcing is a shoaling of the EUC and thermocline, which would presumably enhance the mitigation effect but is not included in this framework.

Observational estimates of historical trends in the essential quantities (EUC velocity and large-scale SST change) provide a very different perspective. Our present best estimates of the historical trend in EUC velocity at 170°W is between 21.4% and 38.7% per century^11^,^13^ (Fig. 4A–C; see Methods). Various instrumental SST data sets can be queried^16^,^17^,^18^, which yield an average trend of 0.25 °C per century at Jarvis and 0.31 °C per century at Baker and Howland. If those trends were to continue, then over 100% of the projected regional-scale warming would be mitigated to the west of Jarvis and the observed cooling trend will continue. Extrapolating observed trends in SST and EUC also results in a mitigation effect of over 100% and 50% at Baker and Howland, respectively. Even if the observed trend to date in EUC velocity is halved and that of SST is doubled, the mitigation effect at Jarvis would be ~30%.

Although sustained *in situ* measurements of comparable quality and spatial distribution have not yet been made at the Gilbert Islands, the relationship between the EUC and δT at the Gilberts developed previously by^5^ can be cast in the same framework (Fig. 4D). Future projections of EUC and large-scale SST at this site from both GCMs and historical extrapolations are qualitatively similar to those for the other three islands. However, the predicted mitigation effect is considerably stronger. This may be due to the unique geography and geometry of the Gilberts, which are larger than the other three islands, forming a chain across the equator with an obvious blockage effect on the EUC^19^ that is striking even in satellite data^20^. The Gilberts will experience a mitigation effect of 28% based on the 14 screened CMIP5 models analyzed, or ~25% based on CMIP3 models^5^. As with Jarvis, historical trends suggest that well over 100% of the warming has been mitigated by circulation over the past century. Of these four islands or island chains in the central equatorial Pacific, Jarvis and the Gilberts clearly hold the greatest potential for future warming mitigation by a strengthening EUC based on historical estimates and future GCM projections.

## Discussion

Stakeholders in climate change are often concerned with impacts not directly simulated by GCMs. This may be due to a simple limitation in spatial resolution, or due to a particular set of physics or phenomena entirely missing from GCMs. The present study is an example of the former limitation, *i.e.*, coral islands and atolls are much too small relative to model grids. Otherwise, GCMs may well simulate the EUC upwelling and resultant changes in island-scale temperatures. Understanding the impacts of climate change on, say, commercial air travel is an example of the latter limitation; airplanes are not emergent features of GCMs, despite GCMs being well suited to simulate the global aviation environment. There is an immediate path forward in these and similar challenges whereby real value can be added to limited GCMs if insight into what is missing from them can be gleaned from observations. Specifically, the observations must bridge the gap between the impact of interest and what GCMs are able and designed to simulate.

To that end, we have brought a robust suite of *in situ* data and state-of-the-art GCMs to bear on the nature of the relationship between global warming, equatorial ocean circulation, and physical stressors at the scale of tiny but crucially important island ecosystems. Jarvis Island and the Gilbert Islands, by accident of geography, appear to stand the best chance to benefit from the mitigating effect of a strengthening EUC. However, the predicted future mitigation effects pale in comparison to those estimated from observed trends—primarily due to the relatively small warming trends to date. Whether observed trends continue or the more severe GCM projections take over, including accelerated warming and more modest EUC strengthening, will play the defining role in shaping marine ecosystems across the equatorial Pacific.

## Methods

### Filling logger gaps with ADCP temperatures

Data gaps in the temperature records from NOAA/CRED temperature/salinity (T/S) loggers at W. Jarvis (<1 year) and E. Baker (~3 years) were filled with temperature sensors onboard the bottom moored Acoustic Doppler Current Profiler (ADCP) instrument packages. The loggers and ADCP were deployed at the same depth and with less than 1 m horizontal displacement. At both sites, temperatures measured by loggers and ADCP are nearly indistinguishable (Fig. S1), justifying their interchangeability. A consistent mean offset of 0.09 °C (0.07 °C) was calculated at W. Jarvis (E. Baker) and applied in the construction of the merged record.

### Using NCEP SST as a proxy for east-side temperatures

Computing the cross-island SST difference (δT) at an island requires knowledge of SST on both the west and east sides of that island. At Jarvis, the west-side record is several years longer than the east-side record and, at Howland, an east-side record does not exist. Fortunately, the gridded (1°) observational SST analysis produced by the NOAA National Centers for Environmental Prediction (NCEP)^21^ based on blending satellite and *in situ* data matches very closely the water temperatures recorded by east-side loggers (Fig. S2). This is because the east side of equatorial islands are largely unaffected by the topographic upwelling present on the west sides. Rather, the east side of an equatorial island is similar to an entire 1° square patch of open ocean. Therefore, the NCEP SST analysis is used in place of east-side loggers so that longer δT records can be utilized and thus facilitate the construction of the most robust possible empirical models of the dependence of δT on the EUC.

### Dependence of results on depth

In general, the reef-building environment is between the surface and roughly 30 m depth. On the west and east sides of Jarvis Islands, we have temperature records available from multiple depths. For our primary results, we use temperatures from 14 m depth at W. Jarvis and 12 m depth from E. Jarvis. These records were selected in order to compare similar depths and utilize the longest records possible. On the east side of an equatorial island, where the logger-based temperature closely follows that of a coarse grid cell (see section b above), temperature is also remarkably constant with depth (Fig. S2). At E. Jarvis, there is very little vertical stratification down to 32 m (Fig. S3). At W. Jarvis, however, there is clearly a tendency for stratification down to 32 m (Fig. S3); most of the stratification appears to occur between 6 m and 14 m. The primary results of this study do not change significantly with different depths, but for completeness the derived empirical models for Jarvis Island are calculated shown separately (Figs 2D and S4).

### Merged EUC record using TAO moorings and SODA reanalysis

The NOAA/PMEL/TAO surface moored ADCP record^12^ extends from 2002–2010 but contains a data gap from mid–2006 through mid–2009 (see Table 1 and Fig. 1). Meanwhile, the pentad resolution SODA reanalysis^13^ extends through 2008. Despite SODA not assimilating any velocity measurements (such as TAO), the two estimates of observed EUC velocity match quite well for the overlapping period 2002–2006 (r = 0.75) (Fig. 1; see also^11^ for a more comprehensive validation). Therefore, to construct a more complete record of EUC velocity at 170W toward more robust empirical models, a merged EUC record was constructed by filling as much of the TAO gap as possible using pentad SODA data after applying a mean offset of −0.01 m s^−1^.

### Empirical model estimation

Records of δT are paired with a merged record of EUC velocity^12^,^13^ (see Table 1) to develop empirical models of the exponential form δT(U) = Ae^BU^ by nonlinear least squares regression (Fig. 2, S4). Models of this form was chosen *a priori* based on the high-resolution numerical ocean model experiment of 5, which suggested a strong exponential relationship between EUC velocity and upwelling strength on the west side of an idealized equatorial island. Experimentation with models of other forms (linear, quadratic, and power) using MATLAB’s Curve Fitting Toolbox yielded inferior goodness-of-fit metrics (particularly linear). At Jarvis, for example, the r^2^ value for the exponential model is 0.45. The r^2^ values for linear, quadratic, and power models are 0.35, 0.43, and 0.43, respectively. The linear model fit is clearly inferior, as it does not capture the obvious nonlinear structure of the covariability between EUC velocity and δT. The quadratic model does yield an r^2^ value close to that of the exponential model, but is unphysical because it predicts an upswing toward higher values of δT as EUC velocity approaches small values. The power model also yields an r^2^ value close to that of the exponential model in this case, and the choice between those two model forms has negligible consequence to the results of the study. At Baker, the r^2^ values for exponential, linear, quadratic, and power models are 0.42, 0.36, 0.41, and 0.41, respectively. At Howland, the r^2^ values for all four types of models are 0.25.

### CMIP5–IPCC AR5 GCM experiments

This study employs the full ensemble archive of 35 global climate models (GCMs) associated with the World Climate Research Programme (WCRP) Coupled Model Intercomparison Project phase 5 (CMIP5) and Intergovernmental Panel on Climate Change (IPCC) Fifth Assessment Report (AR5). The RCP4.5 and RCP8.5 experiments are used, which correspond to 4.5 W/m^2^ and 8.5 W/m^2^ additional radiative forcing by the end of the 21^st^ century, respectively. For further details, see the overview of the CMIP5 experimental design^4^. For calculation of changes in SST and EUC strength over time, linear trends over the full simulations are computed (2006–2100). The complete set of 35 GCMs were screened based on the realism of their climatological EUC simulation compared to observations^7^ and only those 14 models with good simulation of the EUC magnitude, zonal structure, and seasonality were included in the final analysis, although the sensitivity to each screening step is presented in Table 1. A presentation of the results upon which the screening was based (and screened models identified) is presented in Supplementary Data Fig. S5. In addition to computing and presenting multi-model mean results and their spread across all models, the National Center for Atmospheric Research (NCAR) Community Climate System Model version 4 (CCSM4)^15^ is highlighted in the multivariate analysis shown in Fig. 4 simply to demonstrate the results from a single model, including spread within that model’s own ensemble realizations. This model is extensively used and well documented, and plays a leading role in CMIP5/AR5, and previous versions have been featured prominently in the previous CMIP3/AR4. The ocean resolution is 1° (0.27° near the equator) × 60 vertical levels (including 20 10-m thick layers near the surface). While such resolution is not sufficient to resolve small topographic features such as the islands discussed in this paper, this resolution is near the minimum necessary to adequately capture the observed strength of the EUC^14^. For instance, the simulated time-mean velocity of the EUC at 170°W is 0.74 and 0.71 m/s, as compared to 0.75 m/s measured by the TAO mooring (i.e., Fig. 1D).

### Estimated historical EUC trends

Historical trends in EUC strength are estimated based on the Simple Ocean Data Analysis (SODA)^13^. Four trend estimates are calculated by using two different temporal periods (1871–2008 and 1910–2008) and two different SODA versions (v.2.2.4 and v.2.2.6). Differences in the SODA versions are related to different types of observational data assimilated, and v.2.2.6 is an ensemble mean of realizations forced by an ensemble of atmospheric forcing fields, whereas v.2.2.4 is a single realization forced by a single ensemble mean of atmospheric forcing. 1910 is used as a secondary starting time for the trend analysis because that is when the two SODA versions converge and exhibit excellent agreement to the present. Prior to 1910, SODA v.2.2.6 estimates a weaker EUC than v.2.2.4, thus a larger long-term trend for the full period beginning in 1871. In both versions of the SODA reanalysis, the trends computed over the period beginning in 1910 are larger. Accounting for all of the uncertainties (temporal periods, versions, and 95% error bars on the trend calculations), the historical EUC trend is between 17.6% and 43.7% per century.

## Additional Information

**How to cite this article**: Karnauskas, K. B. *et al.* Mitigation of Coral Reef Warming Across the Central Pacific by the Equatorial Undercurrent: A Past and Future Divide. *Sci. Rep.* **6**, 21213; doi: 10.1038/srep21213 (2016).

## Supplementary Material

Supplementary Information

## Figures and Tables

**Table 1:** Effect of screening criteria on projected EUC trends under the RCP8.5 scenario.

Screening criteria applied	Trend	Spread
No screening	9.7%	± 4.2%
Only screen for very weak EUC	10.1%	± 4.7%
Screen for weak and very weak EUC	11.7%	± 4.9%
Screen for weak, very weak, and too strong EUC	11.9%	± 5.1%
Screen for magnitude bias and zonal structure	14.7%	± 5.6%
Screen for magnitude bias, zonal structure, and seasonality	16.3%	± 6.7%

Projected trends and their inter-model spread in EUC strength at 170°W depending on type of screening criteria applied. The criteria applications are generally listed from least stringent (none; 35 models) to most stringent (magnitude bias [either too weak or too strong], zonal structure, and seasonality; 14 models). Trends are expressed in terms of percent change per century, relative to the average over the first decade of the RCP8.5 experiment in each model.
