# Supplementary material for: Mitigation of Coral Reef Warming Across the Central Pacific by the Equatorial Undercurrent: A Past and Future Divide
Source: Sci Rep. 2016 Feb 16;6:21213. doi: 10.1038/srep21213 (PMC4754764; doi:10.1038/srep21213)
Supplement: Supplementary Information [file srep21213-s1.pdf]

Supplementary Information for:

Mitigation of Coral Reef Warming Across the Central Pacific by the Equatorial Undercurrent: A Past and Future Divide

Kristopher B. Karnauskas [1]*, Anne L. Cohen [2], and Jamie M. Gove [3]

[1] Cooperative Institute for Research in Environmental Studies, and Department of Atmospheric and Oceanic Sciences, University of Colorado, Boulder, Colorado, USA

[2] Department of Geology and Geophysics, Woods Hole Oceanographic Institution, Woods Hole, Massachusetts, USA

[3] Ecosystems and Oceanography Division, NOAA Pacific Islands Fisheries Science Center, Honolulu, Hawaii, USA

* Corresponding Author (kristopher.karnauskas@colorado.edu)

Extended Data

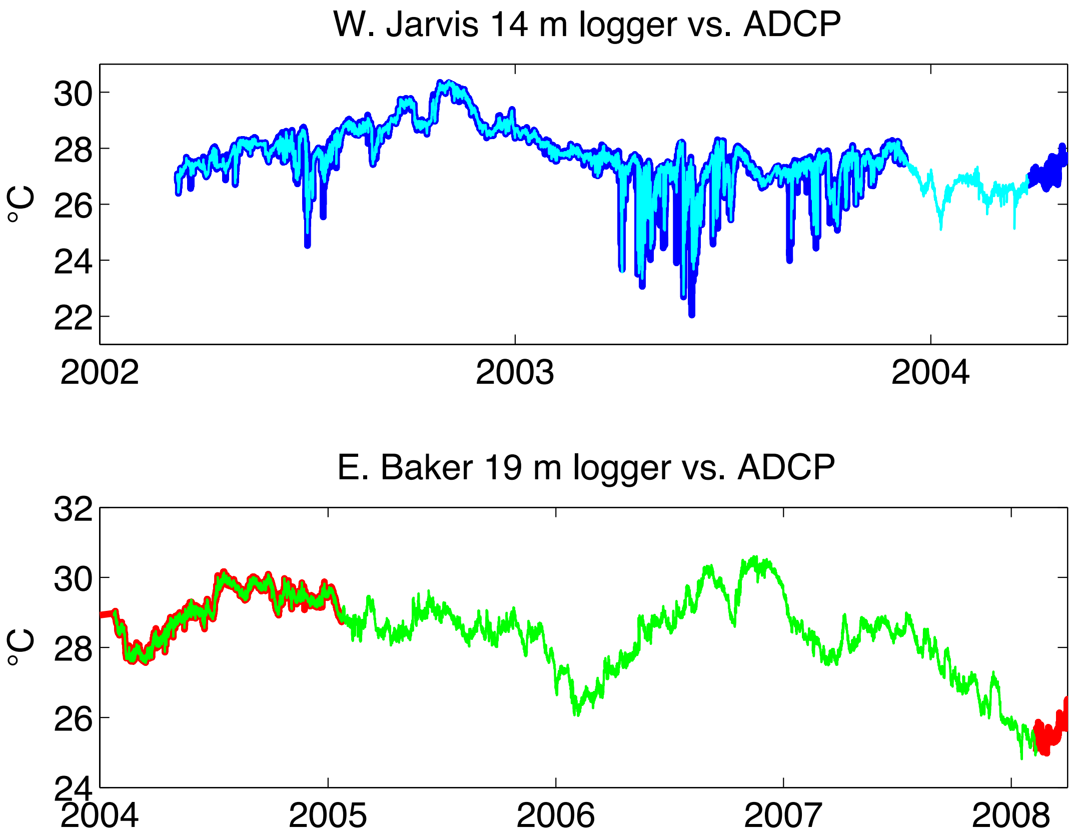

Extended Data Figure 1 | Logger vs. bottom moored ADCP temperatures. Top: 14 m T (°C) on the west side of Jarvis measured by collocated logger (blue) and ADCP package with an offset of -0.089°C (cyan dashed). Bottom: 19 m T (°C) on the east side of Baker measured by collocated logger (red) and ADCP package with an offset of -0.072°C (green dashed). All time series are shown at native sampling resolution; only the overlapping portion of the records are shown. ADCP T records (with offset applied) are used to fill the gaps in logger T records. The gaps to be filled are indicated in both panels by straight-line interpolations.

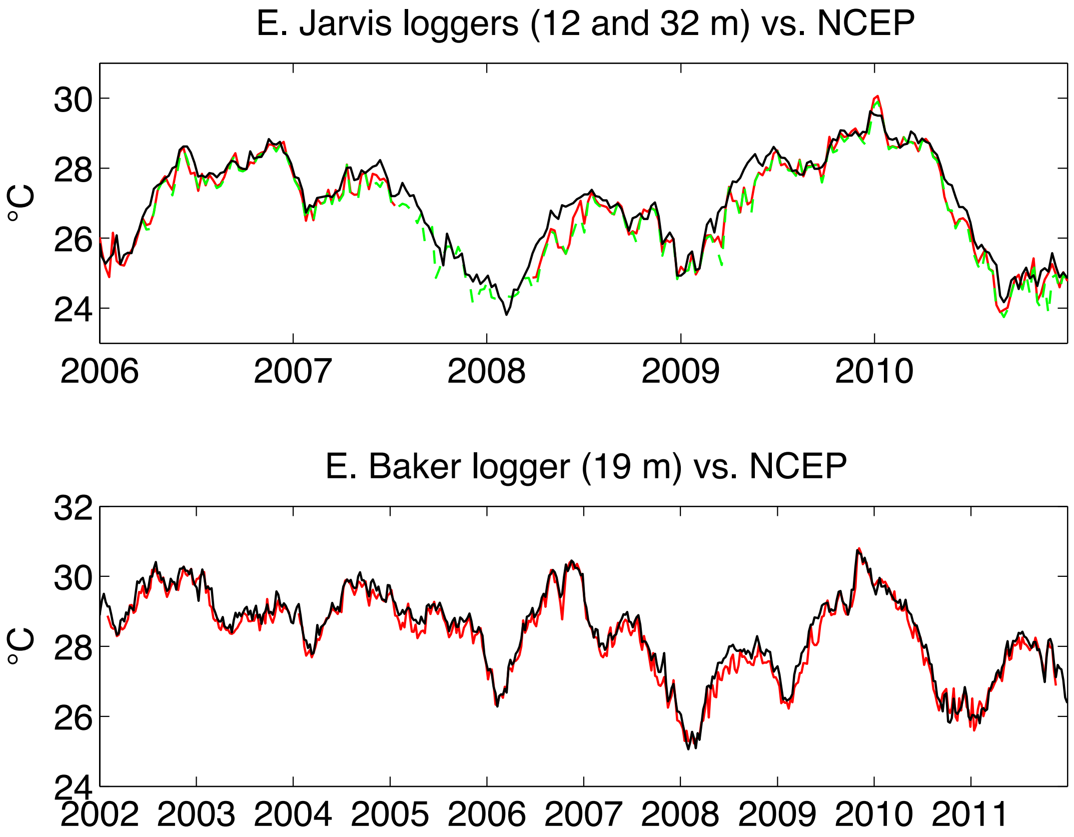

Extended Data Figure 2 | East-side logger temperatures vs. NCEP SST. Top: Temperature (°C) on the east side of Jarvis measured by loggers at 12 m (red) and 32 m (dashed green) and from the NCEP product (black). Bottom: Temperature (°C) on the east side of Baker measured by a logger at 19 m (red) and from the NCEP product (black). All time series are weekly averaged; only the overlapping portion of the records are shown. NCEP SST is used as a proxy for east-side loggers. The linear correlation coefficient between 12 m (19 m) temperature on the east side of Jarvis (Baker) and SST at the nearest NCEP grid cell is 0.974 (0.985).

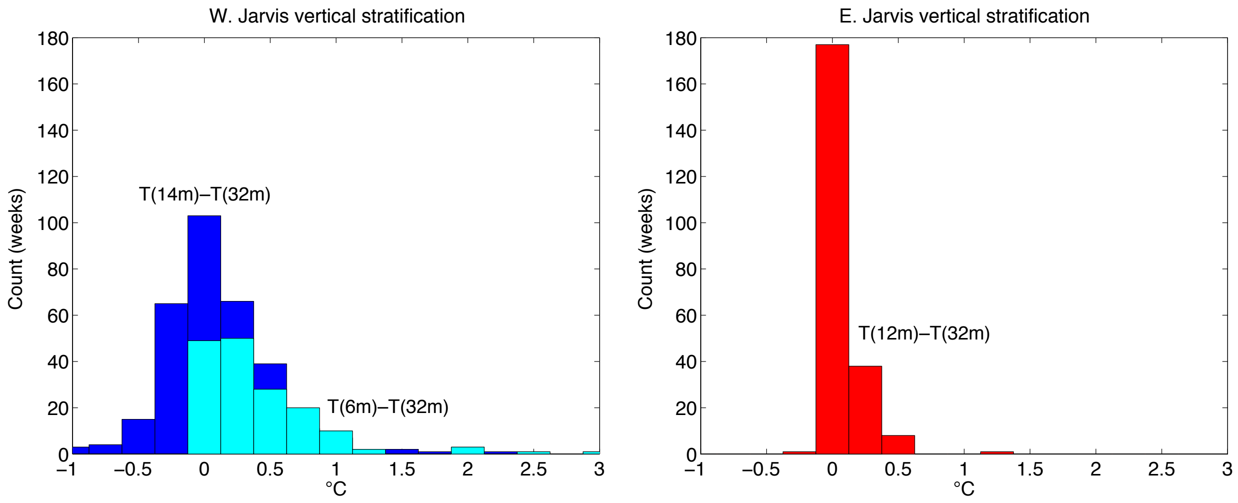

Extended Data Figure 3 | Cross-island gradient in vertical stratification. Left: Histograms of vertical temperature difference (°C) between loggers at 6 m and 32 m (cyan) and between 14 m and 32 m (blue) on the west side of Jarvis Island. Right: As in left but for 12 m and 32 m on the east side of Jarvis. Vertical temperature differences are computed after weekly averaging logger data.

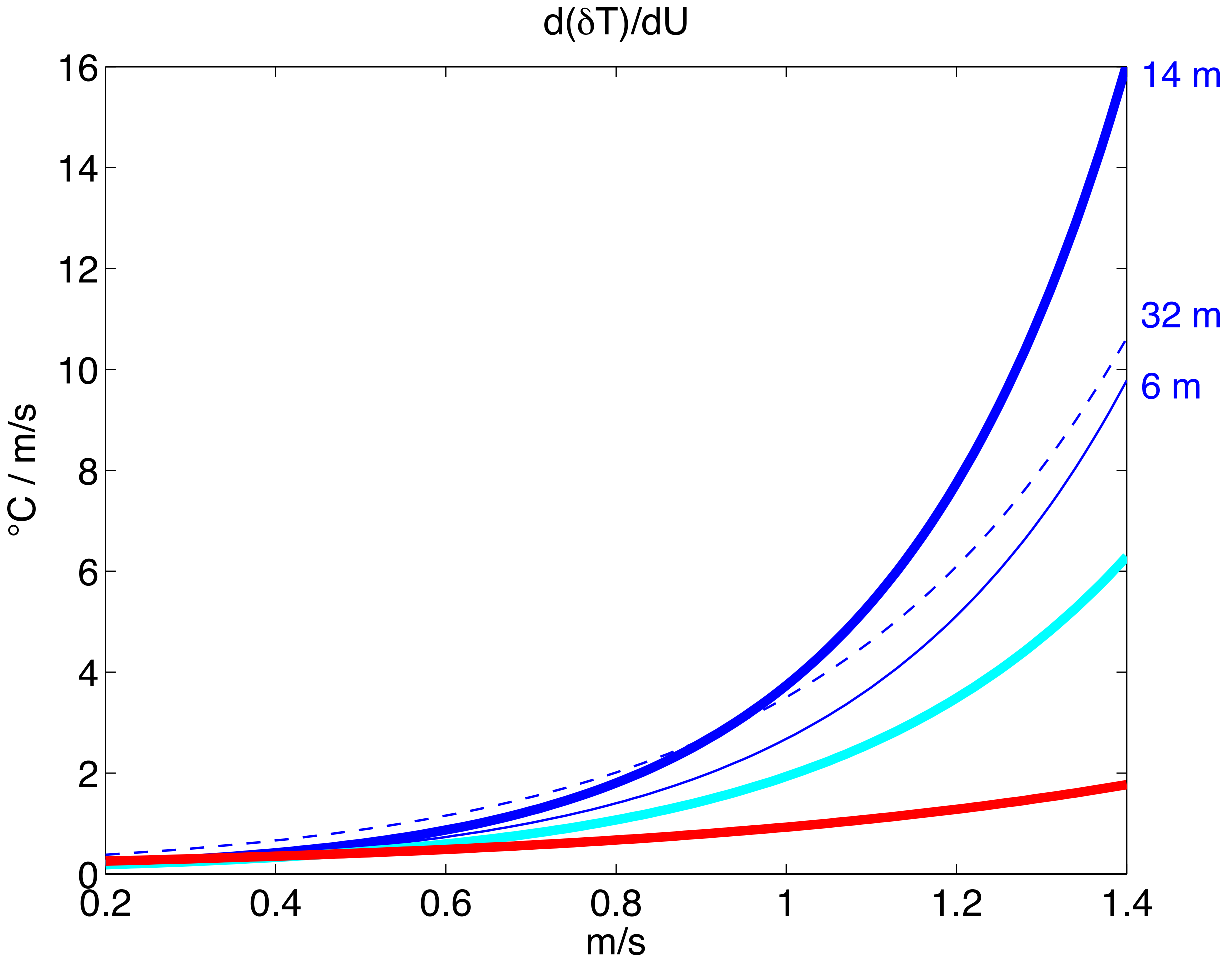

Extended Data Figure 4 | Empirical model in ODE form. As in Fig. 2D but expressed as ordinary differential equations (ODEs) of the form d(δT)/dU for each island (including alternative depths for Jarvis). The lines plotted here are derivatives of the exponential regression model equations shown in Fig. 2A-C.

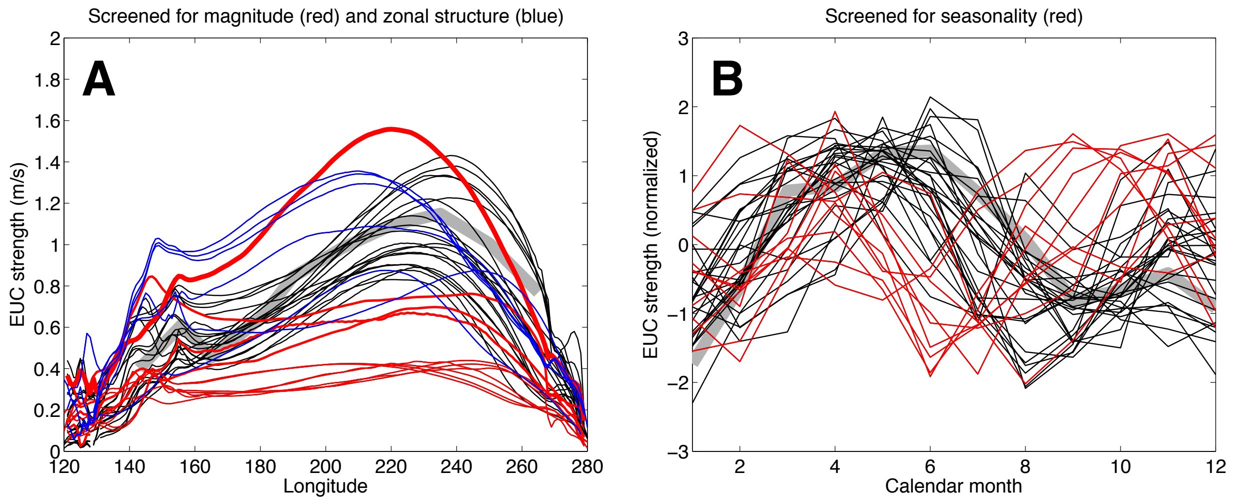

Extended Data Figure 5 | Screening CMIP5 models for EUC simulation. (**A**) As in Fig. 3A but showing all 35 models. Red lines denote biased magnitude and blue lines denote biased zonal structure. (**B**) As in Fig. 3B but showing all 35 models. Red lines denote models with biased seasonality (correlation with observations >0.3).

Param.	Source		T–Range	T–Res.	Coordinates (Site)	H–Res.	Reference	Application in this study
	T/S loggers	Depth						
T	NOAA/CRED	14 m	2002–2012 [1]	Minutes	160.02°W, 0.38°S (W. Jarvis)	Point	Gove *et al.* (2006)	Cross–island δT
T	NOAA/CRED	6 m	2004–2012 [2]	Minutes	160.02°W, 0.38°S (W. Jarvis)	Point	NOAA/CRED	Validation
T	NOAA/CRED	32 m	2006–2012 [3]	Minutes	160.02°W, 0.38°S (W. Jarvis)	Point	NOAA/CRED	Validation
T	NOAA/CRED	12 m	2004–2011 [4]	Minutes	159.98°W, 0.37°S (E. Jarvis)	Point	NOAA/CRED	Cross–island δT
T	NOAA/CRED	32 m	2006–2012 [5]	Minutes	159.98°W, 0.37°S (E. Jarvis)	Point	NOAA/CRED	Validation
T	NOAA/CRED	17 m	2004–2012 [6]	Minutes	176.49°W, 0.19°N (W. Baker)	Point	NOAA/CRED	Cross–island δT
T	NOAA/CRED	19 m	2002–2011 [7]	Minutes	176.47°W, 0.19°N (E. Baker)	Point	NOAA/CRED	Cross–island δT
T	NOAA/CRED	19 m	2004–2012 [8]	Minutes	176.62°W, 0.81°N (W. Howland)	Point	NOAA/CRED	Cross–island δT
	Bottom ADCP	Depth						
T	NOAA/CRED	14 m	2002–2004	Hours	160.02°W, 0.38°S (W. Jarvis)	Point	Gove *et al.* (2006)	Gap filling
T	NOAA/CRED	19 m	2004–2007	Hours	176.47°W, 0.19°N (E. Baker)	Point	NOAA/CRED	Gap filling
	Satellite / *in situ* blend							
T	NOAA/NCEP OI v.2		2002–2012	Weekly	159.5°W, 0.5°S (Jarvis)	1°	Reynolds *et al.* (2002)	Cross–island δT (E. proxy)
T	NOAA/NCEP OI v.2		2002–2012	Weekly	176.5°W, 0.5°N (Baker/Howland)	1°	Reynolds *et al.* (2002)	Cross–island δT (E. proxy)
T	NOAA/NCEP OI v.2		1982–2014	Monthly	Equatorial Pacific	1°	Reynolds *et al.* (2002)	Compute ENSO indices & Climate model validation
	Shipboard ADCP							
U	NOAA/PMEL		1985–2000	Monthly	Equatorial Pacific	~15° [9]	Johnson *et al.* (2002)	Climate model validation
	Surface moored ADCP							
U	NOAA/PMEL/TAO		2002–2010 [10]	Daily	170°W, 0°N	Point	McPhaden *et al.* (1998)	Merged EUC record
	Ocean reanalysis							
U	SODA v.2.2.6		2002–2008 [11]	Pentad	170.25°W, 0.25°S (~TAO 170°W)	0.5°	Carton and Giese (2008)	Merged EUC record
U	SODA v.2.2.6		2002–2008 [11]	Pentad	170.25°W, 0.25°N (~TAO 170°W)	0.5°	Carton and Giese (2008)	Merged EUC record
U	SODA v.2.2.6		1870–2008 [11]	Monthly	Equatorial Pacific	0.5°	Carton and Giese (2008)	Past EUC trend
	Instrumental reconstructions							
T	HadISST1		1870–2012	Monthly	Equatorial Pacific	1°	Rayner *et al.* (2003)	Past SST trends
T	Kaplan SST		1870–2012	Monthly	Equatorial Pacific	5°	Kaplan *et al.* (1998)	Past SST trends
T	NOAA ERSST v.3.b		1870–2012	Monthly	Equatorial Pacific	2°	Smith *et al.* (2008)	Past SST trends

Extended Data Table 1. Observational data sets used in this study. Parameter T indicates water temperature; U indicates zonal ocean velocity. Most data sources except NOAA/CRED Temperature/Salinity (T/S) loggers and bottom moorings begin earlier than date range (T–Range) indicated and used in this study. Time resolution (T–Res.) indicated is the native sampling resolution or averaging interval of the data set as acquired. All logger, satellite, mooring, and reanalysis data were subsequently converted to common weekly averages. Coordinates/site indicates the domain of interest or acquired, not necessarily the spatial domain of the full data set.

[1] Two brief gaps of a few months, ends mid–2012. First gap filled with T recorded by collocated bottom moored ADCP package.
[2] Gap 2007 through 2009, ends mid–2012.
[3] Ends mid–2012.
[4] Water depth 12 m from 2004 through 2010 and 10 m from 2010 onward. Gap mid–2007 through early–2008, ends early–2011.
[5] Ends mid–2012.
[6] Gap during second half 2005, ends mid–2012.
[7] Water depth 19 m from 2002 through 2009 and 11 m from 2010 onward. Gap 2005 through 2007, filled with T recorded by collocated bottom moored ADCP package.
[8] Ends early–2012.
[9] H–Res. here refers to the longitudinal spacing of the meridional ADCP sections made by TAO service cruises, which coincide with the longitudinal spacing of the TAO array itself.
[10] Gap mid–2006 through mid–2009.
[11] Mean of eight ensemble members.

Institution	Model name	Res.	Screening	Reference
BCC	BCC-CSM1.1	0.33		Wu et al. (2014)
BCC	BCC-CSM1.1(m)	0.33		Wu et al. (2014)
BNU	BNU-ESM	0.33		Ji et al. (2014)
CCCMA	CanESM2	0.93	z	Arora et al. (2011)
NCAR	CCSM4	0.27		Gent et al. (2011)
NSF-DOE-NCAR	CESM1(BGC)	0.27		Long et al. (2012)
NSF-DOE-NCAR	CESM1(CAM5)	0.27		Hurrell et al. (2013)
CMCC	CMCC-CM	0.50	s	Scoccimarro et al. (2011)
CMCC	CMCC-CMS	0.50	s	Fogli et al. (2009)
CNRM-CERFACS	CNRM-CM5	0.33		Voldoire et al. (2013)
ICHEC	EC-EARTH	0.33	s	Hazeleger et al. (2010)
LASG-CESS	FGOALS-g2	0.50		Yongqiang et al. (2004)
FIO	FIO-ESM	0.27	z	Qiao et al. (2013)
NOAA-GFDL	GFDL-CM3	0.33		Donner et al. (2011)
NOAA-GFDL	GFDL-ESM2G	0.33	z	Dunne et al. (2013)
NOAA-GFDL	GFDL-ESM2M	0.33	s	Dunne et al. (2013)
NASA-GISS	GISS-E2-H	0.20	m1, s	Schmidt et al. (2006)
NASA-GISS	GISS-E2-H-CC	0.20	m2	Schmidt et al. (2006)
NASA-GISS	GISS-E2-R	1.00	m1	Schmidt et al. (2006)
NASA-GISS	GISS-E2-R-CC	1.00	m1	Schmidt et al. (2006)
NIMR-KMA	HadGEM2-AO	0.33	z	Martin et al. (2011)
MOHC	HadGEM2-CC	0.33	z	Collins et al. (2011)
MOHC	HadGEM2-ES	0.33	z	Collins et al. (2011)
INM	INM-CM4	0.50	s	Voldin et al. (2010)
IPSL	IPSL-CM5A-LR	0.50		Dufresne et al. (2013)
IPSL	IPSL-CM5A-MR	0.50		Dufresne et al. (2013)
IPSL	IPSL-CM5B-LR	0.50		Dufresne et al. (2013)
MIROC	MIROC-ESM	0.50	m1, s	Watanabe et al. (2011)
MIROC	MIROC-ESM-CHEM	0.50	m1	Watanabe et al. (2011)
MIROC	MIROC5	0.50	m1, s	Watanabe et al. (2010)
MPI-M	MPI-ESM-LR	1.50	m1, s	Raddatz et al. (2007)
MPI-M	MPI-ESM-MR	0.40	m3, s	Raddatz et al. (2007)
MRI	MRI-CGCM3	0.50	m2	Yukimoto et al. (2012)
NCC	NorESM1-M	0.27		Iversen et al. (2012)
NCC	NorESM1-ME	0.27		Iversen et al. (2012)

Extended Data Table 2. Global climate models used in this study. Institution and model name of all 35 CMIP5 fully-coupled global climate models used in this study, along with the resolution of the ocean component near the equator (°), the screening criteria that each model did not satisfy (if applicable), and the appropriate reference. Screening criteria codes are: m1, m2, and m3 for very weak, weak and strong EUC bias, respectively; z for zonal structure bias, and s for seasonality bias. See Methods for more details on screening.
